# Editorial: The Emerging Role of Endocannabinoids in Synaptic Plasticity, Reward, and Addiction

**DOI:** 10.3389/fnsyn.2022.898090

**Published:** 2022-05-09

**Authors:** Jeffrey G. Edwards, Luigia Cristino, Dan P. Covey

**Affiliations:** ^1^Department of Cell Biology and Physiology, Neuroscience Center Director, Brigham Young University, Provo, UT, United States; ^2^Institute of Biomolecular Chemistry, Italian National Research Council, Pozzuoli, Italy; ^3^Department of Neuroscience, Lovelace Biomedical Research Institute, Albuquerque, NM, United States

**Keywords:** endocannabinoid, CB1, plasticity, pain, ventral tegmental area, dopamine

## Introduction

Endocannabinoids (eCBs) are lipid-signaling molecules that often work in a retrograde fashion. The most common eCBs are 2-arachidonoylglycerol (2-AG) and anandamide, which bind receptors such as cannabinoid receptor 1 (CB1) and CB2. Endocannabinoid signaling controls synaptic transmission throughout the central nervous system, and is important in modulating activity and behavior in the mesolimbic reward circuit, including the ventral tegmental area (VTA), nucleus accumbens (NAc), and lateral habenula (LHb). In these regions, the eCB system is essential for normal reward learning and for some maladaptive behaviors underlying drug abuse and addiction. Recently identified lipid-signaling eCB-like molecules are also now understood to shape mesolimbic system function and reward-related behaviors.

Further elucidating how the eCB system contributes to reward and addiction is especially pertinent given the recent legalization of medicinal or recreational marijuana throughout the world. The major psychoactive component in marijuana is Δ-9-tetrahydrocannabinol (THC), which binds CB1. Common effects of THC are short-term memory loss, appetite stimulation, and reward. There is still much to investigate concerning THC use, particularly the impact of adolescent use, with a focus on long-term alterations in eCB system function and behavioral changes. Further research is required to clarify the role of the endogenous eCB system, and the effect of exogenous CB1 or CB2-targeting drugs on mesolimbic function, including synaptic plasticity, to support reward behaviors and addiction.

This Research Topic focuses on endogenous eCB system function in the mesolimbic circuit with an emphasis on synaptic plasticity, reward behavior, novel eCB-like molecules, and pain.

## Collection Papers

Dopamine neurons in the VTA and their projections to NAc are essential to reward behavior, motivation, and addiction. The eCB system serves a key role in modulating synaptic transmission in the VTA and NAc to control dopamine signaling and behavior. Covey and Yocky review circuit mechanisms by which eCB signaling modulates dopamine release in the NAc and its implications in motivated behaviors. This review highlights the extraordinary complexity of NAc microcircuitry and discusses emerging techniques capable of dissecting how distinct cell types, their afferent projections, and local neuromodulators interact in the NAc to influence valence-based actions. Oleson et al. further discuss how dopamine-eCB interactions influence goal-directed behaviors. They review a variety of operant behavioral tasks used to dissect how eCBs shape dopamine release to affect distinct and overlapping aspects of motivation, reinforcement, attention, and habit formation.

In addition to canonical eCBs (2-AG and anandamide), eCB-like lipid-signaling molecules have emerged as important neuromodulators regulating dopamine transmission, reward, and addiction. Sagheddu et al. review recently discovered, naturally occurring *N-*acylethanolamines and *N-acyl* amino acids belonging to the lipid signaling system termed endocannabinoidome, and how these can be targeted to treat substance use disorders. Endogenous amides N-Oleoylglycine and N-Oleoylalanine, known to interfere with affective and somatic responses to acute naloxone-precipitated morphine withdrawal, are noted by Rock et al., as ineffective in the establishment of morphine tolerance elicited by morphine. Therefore, the effects of N-Oleoylglycine and N-Oleoylalanine on opiate dependence may be limited to naloxone-precipitated withdrawal effects.

Additional behavioral implications of the eCB system in VTA function include feeding behavior, where it is upregulated by orexin-A-induced enhancement of 2-AG tone and consequent disinhibition of dopaminergic neurons in obese mice, as highlighted by Tunisi et al. Also, Rodríguez-Manzo et al. describe eCB-mediated copulation satiety through changes in glutamate receptors and synaptic plasticity induction in the VTA.

Another important aspect of eCB studies in reward behavior is the long-term impact of adolescent cannabinoid exposure into adulthood, particularly regarding CB2 signaling. Initially, while CB2 receptors were thought to play functional roles only in the periphery, CB2 is expressed in the mesolimbic pathway and controls reward behavior. Ellner et al. demonstrate that CB2 blockade during adolescence has an impact on reward-learning behavior.

The LHb is associated with negative-reward behavior and provides input to the VTA to regulate aversion behavior. Shepard and Nugent provide a perspective on targeting eCB signaling as an intervention to treat mental illness following early life stress that leads to LHb dysfunction, causing disease states such as anxiety and substance use disorder.

Lastly, the eCB system has also been implicated in pro- and anti-nociceptive processes. Paulsen and Burrell highlight how eCBs can support a pro-nociceptive function through activity-dependent sensitization of non-nociceptive fibers through disinhibition of tonic GABA currents. The eCB system is also known to modulate synaptic plasticity to maintain homeostasis of excitatory and inhibitory networks. Pascual et al. used conditional CB1 knock-out in both glutamate and GABA neurons to examine compensatory transcriptomic changes that maintain homeostatic excitatory/inhibitory balance in the CA1 hippocampus. These data may have relevance to pathologies of homeostasis or stress-related disorders.

## Concluding Remarks

Collectively, this Research Topic on eCBs highlights their important role in modulating synaptic transmission throughout the brain to control reward, motivation, pain, and addiction. Work reviewed in this issue discusses recent advancements that have the improved current understanding of the endogenous eCB system function in behavior, and will pave the way for novel therapeutics for psychiatric and neurological disorders.

## Author Contributions

JE, LC, and DC wrote the manuscript. All authors approved the submitted version.

## Funding

JE was supported by NIH grant R15DA049260 and DC by NIH grant R00DA047432.

## Conflict of Interest

The authors declare that the research was conducted in the absence of any commercial or financial relationships that could be construed as a potential conflict of interest.

## Publisher's Note

All claims expressed in this article are solely those of the authors and do not necessarily represent those of their affiliated organizations, or those of the publisher, the editors and the reviewers. Any product that may be evaluated in this article, or claim that may be made by its manufacturer, is not guaranteed or endorsed by the publisher.

